# Economic costs and health-related quality of life outcomes of hospitalised patients with high HIV prevalence: A prospective hospital cohort study in Malawi

**DOI:** 10.1371/journal.pone.0192991

**Published:** 2018-03-15

**Authors:** Hendramoorthy Maheswaran, Stavros Petrou, Danielle Cohen, Peter MacPherson, Felistas Kumwenda, David G. Lalloo, Elizabeth L. Corbett, Aileen Clarke

**Affiliations:** 1 Division of Health Sciences, University of Warwick Medical School, Coventry, United Kingdom; 2 Malawi-Liverpool-Wellcome Trust Clinical Research Programme, Blantyre, Malawi; 3 Department of Public Health and Policy, University of Liverpool, Liverpool, United Kingdom; 4 Department of Clinical Sciences, Liverpool School of Tropical Medicine, Liverpool, United Kingdom; 5 London School of Hygiene and Tropical Medicine, London, United Kingdom; Public Library of Science, UNITED KINGDOM

## Abstract

**Introduction:**

Although HIV infection and its associated co-morbidities remain the commonest reason for hospitalisation in Africa, their impact on economic costs and health-related quality of life (HRQoL) are not well understood. This information is essential for decision-makers to make informed choices about how to best scale-up anti-retroviral treatment (ART) programmes. This study aimed to quantify the impact of HIV infection and ART on economic outcomes in a prospective cohort of hospitalised patients with high HIV prevalence.

**Methods:**

Sequential medical admissions to Queen Elizabeth Central Hospital, Malawi, between June-December 2014 were followed until discharge, with standardised classification of medical diagnosis and estimation of healthcare resources used. Primary costing studies estimated total health provider cost by medical diagnosis. Participants were interviewed to establish direct non-medical and indirect costs. Costs were adjusted to 2014 US$ and INT$. HRQoL was measured using the EuroQol EQ-5D. Multivariable analyses estimated predictors of economic outcomes.

**Results:**

Of 892 eligible participants, 80.4% (647/892) were recruited and medical notes found. In total, 447/647 (69.1%) participants were HIV-positive, 339/447 (75.8%) were on ART prior to admission, and 134/647 (20.7%) died in hospital. Mean duration of admission for HIV-positive participants not on ART and HIV-positive participants on ART was 15.0 days (95%CI: 12.0–18.0) and 12.2 days (95%CI: 10.8–13.7) respectively, compared to 10.8 days (95%CI: 8.8–12.8) for HIV-negative participants. Mean total provider cost per hospital admission was US$74.78 (bootstrap 95%CI: US$25.41-US$124.15) higher for HIV-positive than HIV-negative participants. Amongst HIV-positive participants, the mean total provider cost was US$106.87 (bootstrap 95%CI: US$25.09-US$106.87) lower for those on ART than for those not on ART. The mean total direct non-medical and indirect cost per hospital admission was US$87.84. EQ-5D utility scores were lower amongst HIV-positive participants, but not significantly different between those on and not on ART.

**Conclusions:**

HIV-related hospital care poses substantial financial burdens on health systems and patients; however, per-admission costs are substantially lower for those already initiated onto ART prior to admission. These potential cost savings could offset some of the additional resources needed to provide universal access to ART.

## Introduction

In Eastern and Southern Africa, HIV infection and its associated co-morbidities remain the most common reasons for hospitalisation [1–3]. Up to three quarters of adults admitted for medical reasons are HIV-positive [2], with little change observed since the scale-up of anti-retroviral treatment (ART) began [4, 5]. Hospitals account for a major proportion of health expenditure in the region [6] and reducing the need for hospital care could lead to major cost savings for health systems. However, without a clear understanding of the costs of providing hospital care for people living with HIV, and how this changes with ART [7], decision-makers across the region are unable to include these potential cost savings in assessments of the cost of scaling up ART.

In resource-rich countries, timely initiation of ART in HIV-positive individuals has substantially reduced the need for hospital care and, consequently, the costs of providing HIV care [8, 9]. In Africa, initiation of ART reduces rates and duration of hospitalisations in HIV-positive individuals by up to 70% [10–12], but the degree to which this translates into cost savings for healthcare providers is still uncertain [7]. Timely initiation of ART reduces the risk of opportunistic and TB disease [13], but HIV-positive individuals on ART may still need hospital care, and individuals may incur greater costs during their hospitalisation than those not receiving ART, possibly as a consequence of developing immune constitution syndrome (IRS) [11, 14]. As we move towards immediate initiation of ART for HIV-positive individuals [15], the combined reduction in the risk of developing TB, IRS and other opportunistic illnesses may translate into cost savings. Understanding the impact of timely initiation of ART on the wider health system, especially hospital care, will be essential for budgetary and service planning, and for informing economic evaluations of HIV prevention and treatment interventions.

In this study, we recruited a cohort of adults admitted to the medical wards at Queen Elizabeth Hospital in Blantyre, Malawi. The main aim was to quantify the impact of HIV infection and ART on economic outcomes for adults admitted to these medical wards.

## Methods

### Study design and participants

We undertook a prospective cohort study in Queen Elizabeth Central Hospital (QECH), Blantyre, Malawi, between June and December 2014. We collected medical diagnosis and resource use data, and undertook primary resource-based costing studies to estimate health provider costs. We also investigated the costs incurred by patients and their families as a result of hospitalisation, and evaluated their health-related quality of life (HRQoL) on admission and at regular time intervals thereafter.

QECH is the largest hospital in Malawi, with approximately 1,500 beds and 25,000 adult admissions per year, and an HIV prevalence of approximately 70% amongst medical inpatients [16]. The hospital has a large emergency department where all new patients are triaged and assessed by medical doctors or clinical officers. Clinicians make a preliminary medical diagnosis, and those in need of admission are transferred to one of three medical wards (Male Medical; Female Medical; TB Ward).

Systematic recruitment was used to select every fifth adult (age ≥ 18 years) admission from each of the three ward registers, together with all adults diagnosed with an AIDS defining illness on admission. Participants were approached for informed consent on the first working day after admission. Participants too sick to provide consent were reviewed daily.

A structured questionnaire was used to collect data on the first working day after admission, including socio-demographic data, direct non-medical costs and indirect costs associated with the admission, and health-related quality of life (HRQoL) outcomes. Follow-up questionnaires were administered to participants every three to seven days thereafter, and recorded direct non-medical and indirect costs for the preceding day, and HRQoL on the day of assessment. After discharge or death, a trained study doctor extracted data from the medical notes and drug charts. Primary costing studies were undertaken to estimate direct health provider costs of each hospital admission episode [17, 18].

Ethical approval was obtained from the College of Medicine Ethics Review Committee (P.08/12/1272), University of Malawi, and the University of Warwick Biomedical Research Ethics Committee (REGO-2013-061). All participants provided written (or witnessed thumbprint if illiterate) informed consent.

### Medical diagnosis and resource-use

Data extraction tools and codebooks were developed and piloted to extract the following data from the medical notes: primary medical diagnosis upon discharge or death, HIV status, anti-retroviral drug use, duration of hospital admission, types and numbers of investigations and procedures performed, medications given, and the participant’s outcome (discharged; transferred to another hospital; or died). Coding of the final medical diagnosis upon discharge or death was based on International Classification of Diseases, 9th Revision, Clinical Modification (ICD-9-CM) [19]. Only the primary medical diagnosis that necessitated hospital admission was recorded.

During this study, Malawian national guidelines recommended HIV testing and counselling for all individuals attending or admitted to a health facility, and ART to those who meet eligibility criteria (CD4 count <350 cells/μl; WHO stage 3 or 4; breastfeeding or pregnant). Since August 2016, Malawi has been offering ART to all HIV-positive individuals irrespective of HIV disease stage.

### Direct health provider cost

We identified a list of medical resource inputs (e.g. days of admission; full blood count) from the medical data extracted by the doctors, and then undertook accounting studies to estimate the unit costs for each resource input, and subsequently the total direct health provider cost. For each resource input, we included the cost of: staff salaries; training of staff; consumables and equipment; monitoring and evaluation; and associated overheads. S1 Text provides a detailed description of the costing processes, and how the total direct health provider cost was estimated. The international market price was used for the cost of drugs [20].

### Direct non-medical and indirect cost

The development, language translations and pilot testing of participant questionnaires followed previous procedures [21], with a detailed description provided in S2 Text. The total direct non-medical and indirect cost per participant was estimated for the duration of the hospital admission. This included costs incurred by the participant and their main family member/carer who stayed with them during their hospital admission. The total direct non-medical and indirect cost was estimated by adding the costs on the day of admission, to the average daily cost for each subsequent period between interviews multiplied by the duration of each subsequent period.

The direct non-medical costs included the cost of transportation, food, drinks, toiletries, clothing and other items bought during the hospital admission. For indirect costs, we recorded whether participants or their carers had taken time off work, and if so, the amount of time, and multiplied this by their self-reported income [22]. For self-reported income, we asked participants their average weekly earnings from formal and informal employment, and divided by the average number of days worked per week to estimate average income per day worked. User fees are not charged for care in the hospital, but hospital inpatients may still incur costs of purchasing medications through private providers if there are issues with stocks at the hospital. Participants in this study did not report incurring any such costs.

### Health-related quality of life

The Chichewa version of the EuroQoL EQ-5D-3L [23] was used to assess HRQoL of participants recruited into this study. Participants completed both the descriptive EQ-5D-3L system and the accompanying visual analogue scale (VAS). We derived the EQ-5D utility scores using the Zimbabwean EQ-5D tariff set [24], and report participants’ responses to the visual analogue scale (VAS). The Zimbabwean tariff set generates utility scores ranging between -0.145 and 1.0, with 1.0 corresponding to “perfect health” and 0 representing a health state considered to be equivalent to death. The visual analogue scale is similar to a thermometer, and ranges from 100 (best imaginable health state) to 0 (worst imaginable health state). S3 Text provides a detailed description of procedures used.

### Statistical analysis

All analyses were undertaken in Stata version 13.1 (Stata Corporation, Texas, USA) and R version is 3.2.4 (R Foundation for Statistical Computing, Vienna, Austria). All costs were converted into 2014 US Dollars using market exchange rates and International Dollars using purchasing power parity conversion factors [25, 26]. Principal component analysis was used to generate wealth quintiles by combining socioeconomic variables, which included nine household assets, and home environment variables [27]. The discharge medical diagnosis was coded as the highest level of the four-level ICD-9-CM recorded by the study doctors. Where there were fewer than four participants with the same discharge medical diagnosis, the diagnosis was based upon the next highest level of the four-level ICD-9-CM code recorded by the study doctors.

We estimated the total direct health provider cost, total direct non-medical and indirect cost and total societal cost according to each participant’s discharge medical diagnosis. The total societal cost per participant was estimated by summing the total direct health provider cost, and the total direct non-medical and indirect cost (the latter calculated by summing duration weighted cost estimates from each of the cost assessments). For each of these three cost categories we investigated differences, firstly by HIV status, and secondly by whether or not the participant was on ART at the time of admission. As the cost data was skewed, we used non-parametric bootstrap methods with 1000 bootstrap replications to derive 95% confidence intervals (CI) for mean cost differences for relevant cost categories [28]. In addition, we undertook multivariable analysis to investigate the independent effects of HIV and ART status on these costs. As all participants incurred a cost, and cost data was skewed, we used generalized linear models (GLM) for multivariable analyses of cost data [29]. We ran model diagnostics to determine the optimal choices for the distributional family and link function for these GLM models [30].

For HRQoL assessments, we estimated the EQ-5D utility and VAS scores on admission, on discharge and the change in scores. For the discharge EQ-5D utility score and VAS score, we used the last recorded assessment, and attributed a value of zero for those who died in hospital [31].

We investigated differences in the admission EQ-5D utility and VAS scores by HIV status, and for those who were HIV-positive, by whether or not they were taking ART on admission. In addition, we constructed multivariable models to investigate the independent effects of HIV and ART status on HRQoL assessment on admission. EQ-5D utility and VAS scores were non-normally distributed, skewed and truncated. Therefore, we used non-parametric bootstrap methods, with 1000 bootstrap replications, to derive 95% confidence intervals (CI) for mean differences. For the multivariable analysis, we evaluated four commonly used estimators to analyse these data: ordinary least squares (OLS) regression; Tobit regression, Fractional logit regression, and censored least absolute deviations (CLAD) regression [32–34]. We compared the mean squared error (MSE), mean absolute error (MAE) and the coefficient of determination (r^2^) statistics between the observed and estimated scores for the whole sample, and for sub-groups of the sample to determine the choice of preferred estimator.

For all multivariable analyses of cost and HRQoL outcomes we ran two alternative models, the first adjusted for HIV status, age and sex, and the second additionally adjusted for marital status, educational attainment, income, socio-economic position and the discharge medical diagnosis. We included the discharge medical diagnosis in these models as the aim was to investigate independent associations between HIV and ART status, and cost or HRQoL outcomes.

### Sensitivity analysis

We undertook sensitivity analyses to investigate the impact of using an alternative tariff set to determine EQ-5D utility scores. We used the UK York A1 tariff [35], which has been found to translate health states with ‘severe’ problems in one or more of the five dimensions to lower EQ-5D utility scores than the Zimbabwean tariff [24].

## Results

**Fig 1:**
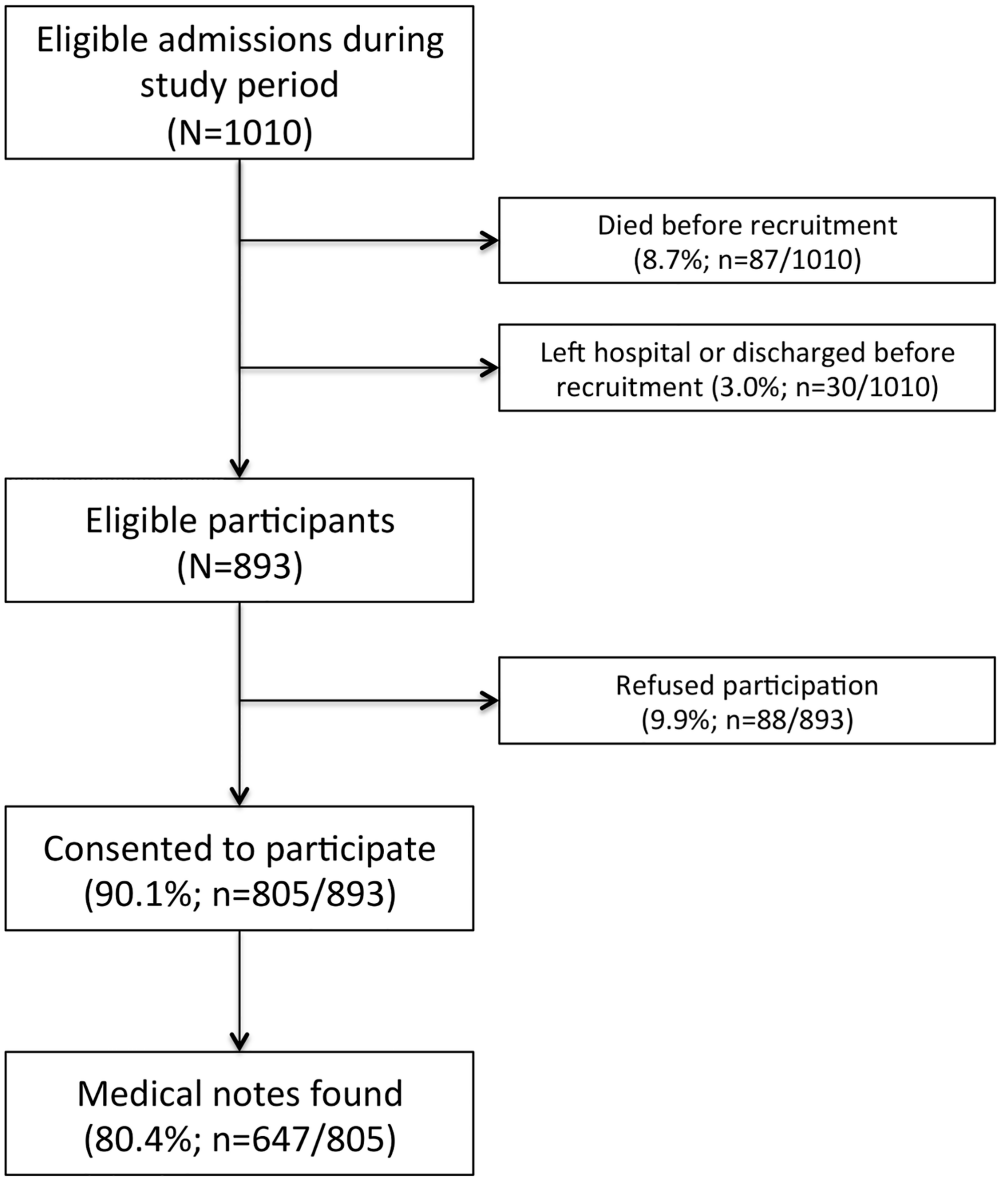
Recruitment of participants. Readmissions were coded as a separate admission.

During the study period 1,010 eligible participants were admitted to the QECH’s adult medical wards (Fig 1). In total, 87 (8.7%) died and 30 (3.0%) left hospital or were discharged before recruitment was possible. Of the remaining 893 eligible participants, 805 (90.1%) consented to participate, and medical notes were found for 647 (80.4%).

Table 1 shows the characteristics of participants by HIV status. Of the 647 participants recruited into the study and for whom the medical notes were found, 134 (20.7%) died in hospital. Overall, 447 (69.1%) were HIV-positive, and 25 (3.9%) had an unknown HIV status. Of those who were HIV-positive, 339 (75.8%) were already on ART on admission. S1–S4 Tables details the health provider per diem cost for each of the three wards; unit costs per dosage of drug dispensed through the QECH’s pharmacy department; and unit costs for investigations and procedures performed.

**Table 1:** Participant characteristics (n = 647).

		HIV negative	HIV positive	HIV status unknown
		n (%)	n (%)	n (%)
**All**		175	447	25
**Sex**	Male	93 (53.1%)	230 (51.5%)	20 (80.0%)
	Female	82 (46.9%)	217 (48.5%)	5 (20.0%)
**Age group (years)**	18–24	31 (17.7%)	38 (8.5%)	5 (20.0%)
	25–34	36 (20.6%)	143 (32.0%)	3 (12.0%)
	35–44	23 (13.1%)	156 (34.9%)	8 (32.0%)
	45+	84 (48.0%)	105 (23.5%)	9 (36.0%)
	Missing	1 (0.6%)	5 (1.1%)	0 (0%)
**Marital status**	Single (never-married)	28 (16.0%)	38 (8.5%)	5 (20.0%)
	Married/cohabiting	101 (57.7%)	243 (54.4%)	10 (10.0%)
	Separated/divorced	12 (6.9%)	85 (19.0%)	2 (8.0%)
	Widower/widow	29 (16.6%)	59 (13.2%)	3 (12.0%)
	Missing	5 (2.9%)	22 (4.9%)	5 (20.0%)
Educational attainment*	Up to standard 8	117 (66.9%)	233 (52.1%)	11 (44.0%)
	Up to form 6	46 (26.3%)	182 (40.7%)	8 (32.0%)
	University or training college	7 (4.0%)	10 (2.2%)	1 (4.0%)
	Missing	5 (2.9%)	22 (4.9%)	5 (20.0%)
Income**	Not working	94 (53.7%)	183 (40.9%)	17 (68.0%)
	Up to 4,000 kwacha/week	21 (12.0%)	62 (13.9%)	3 (12.0%)
	4,000 to 8,000 kwacha/week	18 (10.3%)	59 (13.2%)	2 (8.0%)
	8,000 to 12,000 kwacha/week	7 (4.0%)	32 (7.2%)	0 (0%)
	Over 12,000 kwacha/week	33 (18.9%)	104 (23.3%)	3 (12.0%)
	Missing	2 (1.1%)	7 (1.6%)	0 (0%)
**Employment status**	Formal employment	31 (17.7%)	93 (20.8%)	4 (16.0%)
	Informal employment/Unemployed	50 (28.6%)	161 (36.0%)	10 (40.0%)
	School/University	14 (8.0%)	11 (2.5%)	4 (16.0%)
	Retired	1 (0.6%)	3 (0.7%)	0 (0%)
	Housework	68 (38.9%)	130 (29.1%)	7 (28.0%)
	Sick leave	9 (5.1%)	43 (9.6%)	0 (0%)
	Missing	2 (1.1%)	6 (1.3%)	0 (0%)
Socio-economic position***	Highest quintile	34 (19.4%)	90 (20.1%)	3 (12.0%)
	2nd highest quintile	23 (13.1%)	92 (20.6%)	4 (16.0%)
	Middle quintile	31 (17.7%)	90 (20.1%)	4 (16.0%)
	2nd lowest quintile	34 (19.4%)	82 (18.3%)	3 (12.0%)
	Lowest quintile	45 (25.7%)	67 (15.0%)	6 (24.0%)
	Missing	8 (4.6%)	26 (5.8%)	5 (20.0%)
**ART status**	Not on ART		108 (24.2%)	
	On ART		339 (75.8%)	
**Outcome**	Discharged home alive	151 (86.3%)	342 (76.5%)	20 (80.0%)
	Died as inpatient	24 (13.7%)	105 (23.5%)	5 (20.0%)

ART: Anti-retroviral treatment

*Up to Standard 8 equivalent to completing Primary school; Up to form 6 equivalent to completing Secondary/High school.

**426 Malawian Kwacha = US$1 in 2014

***Socio-economic position estimated though undertaking principal component analysis of responses to asset ownership and housing environment amongst respondents

Table 2 shows the participant characteristics, HIV status and outcomes by the 35 identified discharge medical diagnoses. The three most common reasons for hospital admission were: pneumonia (93/647; 14.4%); septicaemia (58/647; 9.0%); and pulmonary TB (54/647; 8.3%). The mean duration of hospital admission amongst all participants was 12.0 days (95%CI: 11.0–13.1). The mean duration of admission for participants who were HIV-negative, HIV-positive and not on ART, and HIV-positive on ART was 10.8 days (95%CI: 8.8–12.8), 15.0 days (95%CI: 12.0–18.0) and 12.2 days (95%CI: 10.8–13.7), respectively.

**Table 2:** Characteristics of participants by the discharge medical diagnosis (n = 647).

Discharge medical diagnosis	n	Sex	Age	HIV status		Outcome	Days of admission
		Male (n/%)	45+ (n/%)	HIV positive (n/%)	On ART (n/%)	Died (n/%)	Mean (SE)
Pulmonary Tuberculosis	54	39 (72.2%)	10 (18.5%)	47 (87.0%)	34 (63.0%)	14 (25.9%)	23.9 (3.0)
Tuberculosis of the meninges and central nervous system	16	10 (62.5%)	5 (31.3%)	16 (100%)	11 (68.8%)	8 (50.0%)	38.3 (7.3)
Tuberculosis of intestines, peritoneum	9	6 (66.7%)	3 (33.3%)	7 (77.8%)	4 (44.4%)	3 (33.3%)	19.2 (5.6)
Tuberculosis of bones and joint	4	3 (75.0%)	3 (75.0%)	2 (50.0%)	2 (50.0%)	2 (50.0%)	19.0 (5.6)
Tuberculosis of other organs	15	10 (66.7%)	5 (33.3%)	12 (80.0%)	9 (60.0%)	7 (46.7%)	26.2 (8.6)
Miliary Tuberculosis	17	11 (64.7%)	4 (23.5%)	14 (82.4%)	12 (70.6%)	10 (58.8%)	10.7 (1.0)
Tuberculosis—retreatment	6	4 (66.7%)	1 (16.7%)	5 (83.3%)	5 (83.3%)	2 (33.3%)	41.2 (11.8)
Septicaemia*	58	24 (41.4%)	13 (22.4%)	37 (63.8%)	30 (51.7%)	10 (17.2%)	8.4 (1.0)
Candidiasis	6	2 (33.3%)	1 (16.7%)	6 (100%)	5 (83.3%)	1 (16.7%)	5.7 (1.7)
Cryptococcal meningitis	36	27 (75.0%)	3 (8.3%)	36 (100%)	27 (75.0%)	9 (25.0%)	15.9 (1.8)
Viral infection	8	4 (50.0%)	1 (12.5%)	8 (100%)	6 (75.0%)	4 (50.0%)	15.3 (3.9)
Pneumocystis Jivorecii pneumonia	9	4 (44.4%)	1 (11.1%)	9 (100%)	3 (33.3%)	2 (22.2%)	13.4 (1.9)
Malaria	13	3 (23.1%)	3 (23.1%)	10 (76.9%)	6 (46.2%)	1 (7.7%)	5.2 (1.6)
Kaposi’s sarcoma	20	16 (80.0%)	2 (10.0%)	20 (100%)	17 (85.0%)	5 (25.0%)	9.1 (1.2)
Neoplasms—excluding Kaposi's	7	4 (57.1%)	3 (42.9%)	3 (42.9%)	2 (28.6%)	1 (14.3%)	15.6 (2.1)
Diabetes mellitus without complications	5	0 (0.0%)	3 (60.0%)	0 (0%)	0 (0%)	0 (0%)	3.8 (1.2)
Diabetes mellitus with complications	9	5 (55.6%)	6 (66.7%)	1 (11.1%)	1 (11.1%)	0 (0%)	8.2 (1.2)
Anaemia	35	14 (40.0%)	11 (31.4%)	26 (74.3%)	24 (68.6%)	6 (17.1%)	9.4 (1.3)
Mental health disorders	9	6 (66.7%)	2 (22.2%)	2 (22.2%)	1 (11.1%)	0 (0%)	6.6 (1.9)
Meningitis**	37	11 (29.7%)	11 (29.7%)	26 (70.3%)	19 (51.4%)	5 (13.5%)	9.2 (0.8)
Epilepsy; Convulsions	10	5 (50.0%)	2 (20.0%)	3 (30.0%)	3 (30.0%)	1 (10.0%)	6.3 (0.9)
Other neurological problems	16	12 (75.0%)	4 (25.0%)	8 (50.0%)	8 (50.0%)	1 (6.3%)	10.3 (2.6)
Cerebrovascular disease	25	12 (48.0%)	16 (64.0%)	10 (40.0%)	6 (24.0%)	2 (8.0%)	8.6 (1.1)
Hypertension	7	5 (71.4%)	5 (71.4%)	2 (28.6%)	1 (14.3%)	2 (28.6%)	11.1 (4.4)
Congestive heart failure; non-hypertensive	15	6 (40.0%)	12 (80.0%)	2 (13.3%)	0 (0.0%)	5 (33.3%)	9.4 (2.1)
Other cardiovascular problems	12	4 (33.3%)	8 (66.7%)	5 (41.7%)	4 (33.3%)	2 (16.7%)	10.2 (3.7)
Pneumonia**	93	51 (54.8%)	23 (24.7%)	74 (79.6%)	56 (60.2%)	13 (14.0%)	7.5 (0.9)
Other respiratory problems	11	3 (27.3%)	6 (54.6%)	5 (45.5%)	4 (36.4%)	1 (9.1%)	9.9 (2.4)
Acute—Intestinal infection	10	10 (100%)	4 (40.0%)	6 (60.0%)	5 (50.0%)	1 (10.0%)	12.6 (3.5)
Chronic—Intestinal infection	14	5 (35.7%)	4 (28.6%)	11 (78.6%)	10 (71.4%)	4 (28.6%)	6.7 (1.4)
Upper gastrointestinal disorders	11	2 (18.2%)	4 (36.4%)	8 (72.7%)	7 (63.6%)	2 (18.2%)	5.7 (0.6)
Liver disease	14	8 (57.1%)	4 (28.6%)	9 (64.3%)	6 (42.9%)	6 (42.9%)	10.0 (1.9)
Diseases of the genitourinary system	18	7 (38.9%)	8 (44.4%)	14 (77.8%)	9 (50.0%)	3 (16.7%)	7.7 (1.3)
Diseases of the musculoskeletal system	6	5 (83.3%)	4 (66.7%)	2 (33.3%)	1 (16.7%)	0 (0%)	15.0 (4.2)
Other problems (<5 cases)	12	5 (41.7%)	3 (25.0%)	1 (8.3%)	1 (8.3%)	1 (8.3%)	7.3 (0.9)

*Except in Labour

**Except that caused by TB or Cryptococcal

**Fig 2:**
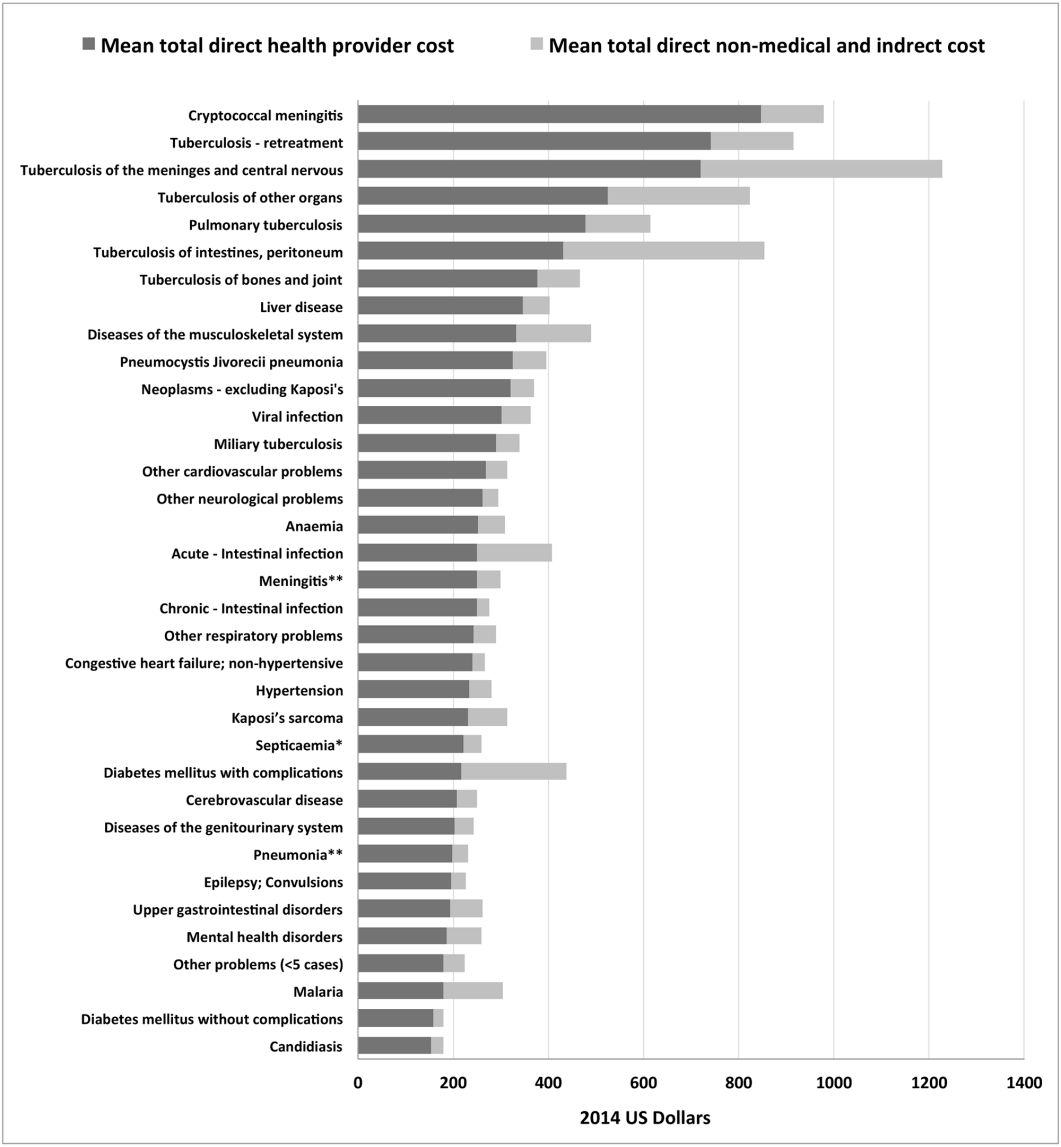
Mean total costs by discharge medical diagnosis. *Except in Labour **Except that caused by TB or Cryptococcal Total societal cost equates to the total direct health provider cost plus the total direct non-medical and indirect cost.

The mean total health provider cost per individual hospital admission, and the mean average daily cost, were US$313.65 (INT$788.83) and US$32.14 (INT$80.77), respectively (Table 3). Ward costs accounted for 61.2%, investigations and medical procedures accounted for 35.5%, and drugs accounted for 3.6% of the total International Dollar costs. The three discharge medical diagnoses associated with the highest mean total health provider costs were: cryptococcal meningitis (US$846.24); retreatment for TB (US$741.14); and TB of the meninges and central nervous system (US$721.02) (Fig 2).

**Table 3:** Total direct health provider costs by discharge medical diagnosis (n = 647).

Discharge medical diagnosis		Total health provider cost		Average daily cost		Mean proportion of total heath provider cost		
		2014 US Dollars	2014 INT Dollars	2014 US Dollars	2014 INT Dollars	% Drugs	% Investigations & Procedures	% Ward stay
	N	Mean (SE)	Mean (SE)	Mean (SE)	Mean (SE)			
**All**	**647**	**313.65 (12.2)**	**788.83 (27.7)**	**32.14 (0.9)**	**80.77 (1.9)**	**3.6**	**35.5**	**61.2**
Pulmonary tuberculosis	54	477.57 (51.5)	1252.59 (137.2)	23.67 (1.0)	61.11 (2.6)	3.4	26.2	70.8
Tuberculosis of the meninges and central nervous system	16	721.02 (117.3)	1891.95 (306.6)	32.93 (5.7)	87.60 (15.5)	2.9	33.0	64.6
Tuberculosis of intestines, peritoneum	9	429.92 (97.2)	1119.28 (252.3)	26.24 (3.6)	68.23 (9.3)	3.1	29.3	67.9
Tuberculosis of bones and joint	4	376.51 (106.7)	1016.96 (288.9)	19.82 (0.9)	53.62 (2.03)	1.7	19.8	78.9
Tuberculosis of other organs	15	523.78 (138.1)	1386.15 (378.5)	25.95 (2.5)	66.52 (6.0)	3.6	29.1	67.6
Miliary tuberculosis	17	289.61 (25.5)	753.68 (66.9)	28.16 (1.9)	73.22 (4.8)	3.1	35.8	61.5
Tuberculosis—retreatment	6	741.14 (203.4)	1943.75 (535.0)	20.24 (2.1)	52.69 (5.4)	3.6	15.4	81.7
Septicaemia*	58	222.13 (18.0)	580.97 (47.3)	33.71 (2.3)	87.84 (6.0)	2.4	40.4	57.5
Candidiasis	6	153.08 (43.1)	395.98 (113.9)	31.12 (6.0)	77.70 (11.9)	2.8	35.5	62.2
Cryptococcal meningitis	36	846.24 (101.9)	1583.26 (138.6)	64.26 (9.5)	114.49 (10.9)	20.9	32.6	46.8
Viral infection	8	300.80 (70.1)	808.37 (192.2)	25.34 (5.7)	66.71 (14.6)	1.8	21.4	77.4
Pneumocystis Jivorecii pneumonia	9	325.56 (28.3)	849.35 (75.5)	26.79 (3.0)	69.55 (7.6)	2.9	29.9	67.3
Malaria	13	179.01 (36.0)	439.87 (94.3)	44.78 (6.7)	106.46 (14.4)	7.2	44.2	48.8
Kaposi’s sarcoma	20	230.99 (25.8)	609.69 (69.4)	28.99 (2.7)	75.55 (6.7)	2.5	35.5	62.5
Neoplasms—excluding Kaposi's	7	320.31 (36.8)	839.86 (99.2)	21.08 (0.8)	54.88 (1.5)	2.6	19.1	78.4
Diabetes mellitus without complications	5	158.72 (40.3)	403.59 (106.2)	46.07 (8.6)	116.25 (20.7)	5.5	50.7	43.8
Diabetes mellitus with complications	9	217.13 (31.1)	574.67 (82.3)	30.54 (4.6)	80.10 (11.4)	3.1	36.7	60.2
Anaemia	35	251.79 (25.8)	678.01 (70.9)	33.43 (4.1)	89.61 (11.5)	1.7	39.7	58.9
Mental health disorders	9	186.64 (38.4)	496.94 (103.3)	33.14 (4.2)	87.91 (10.8)	2.0	44.5	53.5
Meningitis**	37	250.76 (17.8)	646.86 (45.9)	30.76 (1.8)	79.23 (4.6)	3.2	37.2	59.9
Epilepsy; Convulsions	10	195.01 (17.1)	501.68 (44.4)	34.84 (3.9)	88.86 (8.9)	2.6	44.5	53.0
Other neurological problems	16	261.58 (48.6)	682.28 (127.9)	32.85 (3.3)	86.00 (8.5)	1.5	42.1	56.6
Cerebrovascular disease	25	207.26 (25.2)	551.48 (64.5)	26.66 (1.6)	71.18 (4.2)	1.9	32.5	65.8
Hypertension	7	234.21 (79.1)	633.97 (212.2)	25.47 (3.6)	68.18 (8.6)	1.7	31.6	66.8
Congestive heart failure; non-hypertensive	15	239.51 (47.6)	646.48 (132.4)	27.82 (2.7)	74.61 (7.3)	1.4	33.4	65.2
Other cardiovascular problems	12	269.03 (81.0)	702.02 (205.2)	29.99 (2.3)	79.42 (6.0)	2.7	38.4	59.1
Pneumonia**	93	198.77 (14.4)	517.30 (39.4)	30.81 (0.9)	79.04 (2.2)	2.2	39.7	58.4
Other respiratory problems	11	242.98 (56.0)	641.18 (148.1)	25.93 (2.2)	68.01 (5.3)	2.6	29.5	68.1
Acute—Intestinal infection	10	250.82 (53.1)	667.67 (147.6)	23.10 (1.8)	60.53 (4.6)	3.3	23.7	73.5
Chronic—Intestinal infection	14	249.26 (61.0)	658.73 (166.3)	50.21 (18.8)	133.16 (52.0)	2.7	43.5	54.3
Upper gastrointestinal disorders	11	193.04 (46.0)	508.40 (129.3)	32.93 (5.0)	86.30 (14.1)	2.2	39.2	59.0
Liver disease	14	345.84 (103.9)	940.74 (287.6)	31.82 (3.2)	85.74 (8.8)	1.4	42.7	56.1
Diseases of the genitourinary system	18	202.25 (24.5)	537.57 (67.0)	29.02 (1.7)	76.68 (4.2)	2.1	38.1	60.1
Diseases of the musculoskeletal system	6	332.33 (79.2)	875.32 (203.1)	23.59 (1.4)	62.42 (3.9)	1.9	28.1	70.1
Other problems (<5 cases)	12	179.93 (23.3)	477.10 (58.2)	25.13 (1.5)	66.92 (3.6)	1.9	30.6	67.6

*Except in Labour

**Except that caused by TB or Cryptococcal

Table 4 shows the mean total direct non-medical and indirect costs, and the mean total societal costs, for all participants by discharge medical diagnosis. The mean total direct non-medical and indirect cost per hospital admission was US$87.84 (INT$243.99). The mean total societal cost per hospital admission was US$401.48 (INT$1032.82). The three discharge medical diagnoses associated with the highest mean total societal costs were: TB of the meninges and central nervous system (US$1228.38); cryptococcal meningitis (US$977.75); and retreatment for TB (US$915.32).

**Table 4:** Total direct non-medical and indirect, and societal costs by discharge medical diagnosis (n = 647).

Discharge medical diagnosis		Total direct non-medical and indirect cost		Total societal cost	
		2014 US Dollars	2014 INT Dollars	2014 US Dollars	2014 INT Dollars
	N	Mean (SE)	Mean (SE)	Mean (SE)	Mean (SE)
**All**	**647**	**87.84 (10.2)**	**243.99 (28.3)**	**401.48 (18.8)**	**1032.82 (48.1)**
Pulmonary tuberculosis	54	135.59 (29.7)	376.64 (82.5)	613.16 (68.6)	1629.23 (185.1)
Tuberculosis of the meninges and central nervous system	16	507.36 (205.6)	1409.33 (571.0)	1228.38 (284.7)	3301.29 (771.2)
Tuberculosis of intestines, peritoneum	9	424.13 (336.4)	1178.14 (934.4)	854.05 (431.0)	2297.42 (1179.0)
Tuberculosis of bones and joint	4	90.43 (34.9)	251.20 (97.1)	466.94 (134.3)	1268.16 (365.8)
Tuberculosis of other organs	15	299.43 (189.7)	831.74 (526.9)	823.21 (318.7)	2217.89 (882.0)
Miliary tuberculosis	17	48.55 (12.6)	134.86 (34.9)	338.16 (30.3)	888.54 (80.2)
Tuberculosis—retreatment	6	174.18 (130.8)	483.83 (363.4)	915.32 (280.4)	2427.58 (744.8)
Septicaemia*	58	38.05 (8.2)	105.68 (22.9)	260.17 (24.2)	686.65 (64.6)
Candidiasis	6	25.86 (20.0)	71.84 (55.6)	178.94 (58.8)	467.81 (156.7)
Cryptococcal meningitis	36	131.50 (44.1)	365.28 (122.4)	977.75 (113.6)	1948.54 (206.2)
Viral infection	8	61.51 (32.9)	170.87 (91.4)	362.32 (89.5)	979.24 (245.7)
Pneumocystis Jivorecii pneumonia	9	69.59 (28.2)	193.30 (78.2)	395.15 (50.6)	1042.65 (139.6)
Malaria	13	124.91 (116.9)	346.97 (324.7)	303.92 (119.3)	786.85 (328.1)
Kaposi’s sarcoma	20	81.66 (21.5)	226.84 (59.6)	312.65 (43.2)	836.53 (118.3)
Neoplasms—excluding Kaposi's	7	49.03 (15.6)	136.19 (43.3)	369.34 (45.8)	976.06 (126.0)
Diabetes mellitus without complications	5	21.63 (10.6)	60.07 (29.6)	180.34 (48.8)	463.66 (129.9)
Diabetes mellitus with complications	9	220.34 (145.4)	612.05 (404.0)	437.47 (157.4)	1186.72 (435.9)
Anaemia	35	57.20 (10.5)	158.90 (29.2)	308.99 (33.0)	836.91 (90.9)
Mental health disorders	9	72.97 (30.3)	202.69 (84.1)	259.61 (53.0)	699.64 (142.8)
Meningitis**	37	49.54 (10.3)	137.61 (28.7)	300.30 (23.1)	784.47 (61.4)
Epilepsy; Convulsions	10	30.86 (19.8)	85.71 (55.0)	225.86 (27.9)	587.39 (75.7)
Other neurological problems	16	31.98 (9.3)	88.84 (25.9)	293.56 (56.1)	771.12 (149.1)
Cerebrovascular disease	25	42.98 (13.6)	119.39 (37.7)	250.24 (34.4)	670.87 (89.6)
Hypertension	7	46.52 (24.1)	129.23 (66.9)	280.73 (102.3)	763.20 (276.4)
Congestive heart failure; non-hypertensive	15	28.02 (7.3)	77.83 (20.2)	267.53 (49.3)	724.31 (136.6)
Other cardiovascular problems	12	44.31 (16.6)	123.08 (46.0)	313.34 (93.1)	825.10 (239.4)
Pneumonia**	93	33.36 (5.6)	92.65 (15.6)	232.12 (19.0)	609.95 (52.4)
Other respiratory problems	11	46.70 (18.5)	129.71 (51.4)	289.67 (59.7)	770.89 (158.8)
Acute—Intestinal infection	10	155.70 (53.4)	432.49 (148.3)	406.52 (94.4)	1100.17 (262.8)
Chronic—Intestinal infection	14	26.29 (9.6)	73.03 (26.7)	275.55 (64.2)	731.76 (175.5)
Upper gastrointestinal disorders	11	68.22 (41.2)	189.49 (114.5)	261.26 (58.0)	697.89 (162.8)
Liver disease	14	56.37 (24.4)	156.57 (67.7)	402.21 (104.1)	1097.31 (288.1)
Diseases of the genitourinary system	18	39.71 (13.3)	110.31 (37.0)	241.96 (30.3)	647.87 (82.4)
Diseases of the musculoskeletal system	6	158.44 (93.6)	440.11 (260.1)	490.77 (111.2)	1315.43 (301.6)
Other problems (<5 cases)	12	44.46 (16.4)	123.50 (45.6)	224.39 (34.0)	600.60 (86.8)

*Except in Labour

**Except that caused by TB or Cryptococcal

**Fig 3:**
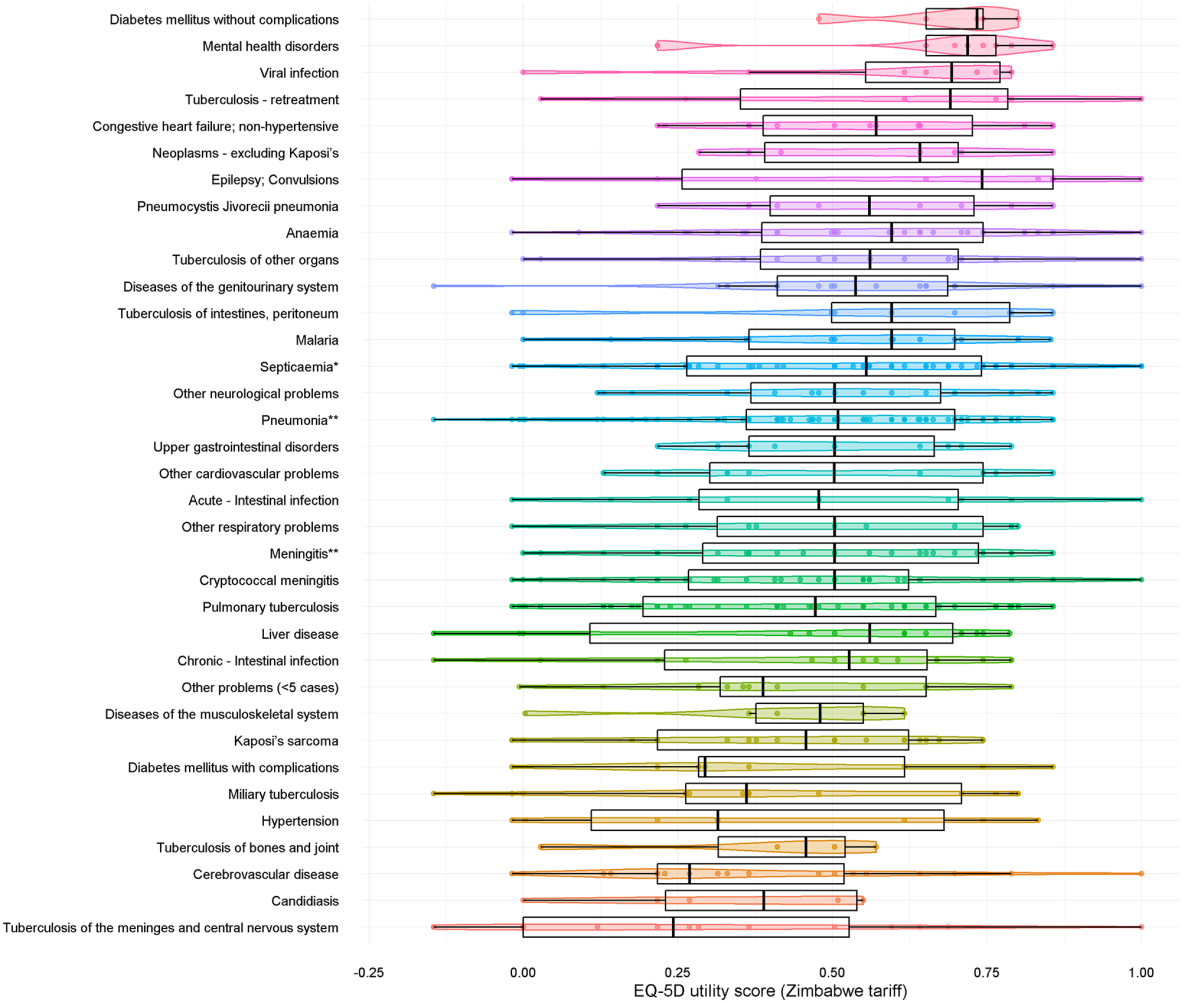
Frequency, distribution and density of EQ-5D utility scores by medical diagnosis. *Except in Labour **Except that caused by TB or Cryptococcal.

The EQ-5D utility and VAS scores for all participants, and by discharge medical diagnosis, are shown in Table 5. For all participants, the mean EQ-5D utility score and VAS score on admission was 0.483 (SE: 0.01) and 52.8 (SE: 0.8), respectively (Table 5). The three discharge medical diagnoses associated with the lowest EQ-5D utility scores on admission were TB of the meninges and central nervous system, candidiasis and cerebrovascular disease (Fig 3). For all participants, the mean change in EQ-5D utility and VAS scores was 0.020 (SE: 0.01) and 0.4 (SE: 1.2), respectively. The mean change in EQ-5D utility score, derived using the UK tariff set, was 0.116 (SE: 0.02) (S5 Table).

**Table 5:** Health-related quality of life outcomes by discharge medical diagnosis (n = 640).

Discharge medical diagnosis	N	EQ-5D utility scores (Zimbabwean tariff)			VAS scores		
		On admission	Last recorded	Change	On admission	Last recorded	Change
		Mean (SE)	Mean (SE)	Mean (SE)	Mean (SE)	Mean (SE)	Mean (SE)
**All**	**640**	**0.483 (0.01)**	**0.503 (0.01)**	**+0.020 (0.01)**	**52.8 (0.8)**	**53.2 (1.3)**	**+0.4 (1.2)**
Pulmonary tuberculosis	54	0.445 (0.04)	0.486 (0.05)	+0.041 (0.04)	55.1 (3.0)	55.4 (5.0)	+0.2 (4.4)
Tuberculosis of the meninges and central nervous system	16	0.275 (0.08)	0.304 (0.09)	+0.030 (0.12)	41.3 (7.2)	37.4 (10.1)	-3.9 (8.6)
Tuberculosis of intestines, peritoneum	9	0.524 (0.11)	0.430 (0.11)	-0.094 (0.07)	50.0 (7.5)	42.2 (12.1)	-7.8 (11.4)
Tuberculosis of bones and joint	4	0.379 (0.12)	0.277 (0.16)	-0.101 (0.10)	65.0 (9.6)	45.0 (26.3)	-20.0 (17.3)
Tuberculosis of other organs	15	0.542 (0.08)	0.376 (0.10)	-0.166 (0.10)	51.0 (6.1)	41.3 (10.7)	-9.7 (10.5)
Miliary tuberculosis	17	0.393 (0.07)	0.185 (0.07)	-0.208 (0.07)	38.5 (4.4)	20.9 (7.5)	-17.6 (8.2)
Tuberculosis—retreatment	6	0.577 (0.15)	0.545 (0.17)	-0.032 (0.08)	73.3 (7.6)	54.2 (17.6)	-19.2 (19.0)
Septicaemia*	58	0.512 (0.04)	0.577 (0.04)	+0.064 (0.04)	53.0 (2.9)	55.2 (4.2)	+2.1 (3.8)
Candidiasis	6	0.349 (0.09)	0.424 (0.10)	+0.074 (0.08)	48.3 (11.7)	50.0 (11.8)	+1.7 (1.7)
Cryptococcal meningitis	36	0.478 (0.04)	0.474 (0.06)	-0.004 (0.06)	56.4 (3.3)	52.2 (5.6)	-4.1 (6.1)
Viral infection	8	0.589 (0.10)	0.395 (0.15)	-0.195 (0.12)	56.3 (9.8)	41.9 (15.9)	-14.4 (12.2)
Pneumocystis Jivorecii pneumonia	8	0.559 (0.08)	0.501 (0.15)	-0.058 (0.16)	55.0 (8.0)	58.8 (13.3)	+3.8 (11.0)
Malaria	13	0.521 (0.07)	0.514 (0.08)	-0.006 (0.03)	53.2 (6.6)	53.9 (6.5)	+0.8 (11.0)
Kaposi’s sarcoma	20	0.415 (0.06)	0.402 (0.06)	-0.014 (0.05)	48.3 (3.5)	46.5 (7.2)	+0.8 (1.4)
Neoplasms—excluding Kaposi's	7	0.567 (0.08)	0.342 (0.15)	-0.225 (0.13)	52.9 (5.2)	42.1 (11.0)	-10.7 (12.8)
Diabetes mellitus without complications	5	0.682 (0.06)	0.815 (0.05)	+0.133 (0.08)	73.4 (8.1)	80.4 (9.0)	+7.0 (3.7)
Diabetes mellitus with complications	9	0.405 (0.09)	0.443 (0.09)	+0.038 (0.07)	54.4 (3.4)	57.2 (5.7)	+2.8 (3.6)
Anaemia	35	0.558 (0.04)	0.586 (0.06)	+0.028 (0.05)	52.5 (3.2)	59.4 (5.4)	+7.0 (5.3)
Mental health disorders	9	0.629 (0.08)	0.705 (0.07)	+0.076 (0.09)	61.1 (4.8)	67.2 (6.1)	+6.1 (4.2)
Meningitis**	36	0.484 (0.04)	0.611 (0.05)	+0.126 (0.05)	49.9 (3.5)	59.3 (4.8)	+9.4 (3.8)
Epilepsy; Convulsions	10	0.561 (0.12)	0.560 (0.14)	-0.002 (0.05)	57.0 (6.3)	64.0 (8.7)	+7.0 (5.0)
Other neurological problems	15	0.506 (0.06)	0.524 (0.07)	+0.018 (0.06)	55.3 (4.1)	57.5 (6.8)	+2.2 (5.7)
Cerebrovascular disease	23	0.359 (0.05)	0.438 (0.07)	+0.078 (0.04)	50.7 (4.9)	54.3 (5.3)	+3.7 (5.5)
Hypertension	7	0.387 (0.13)	0.419 (0.14)	+0.032 (0.11)	58.6 (2.6)	50.7 (13.8)	-7.9 (15.9)
Congestive heart failure; non-hypertensive	15	0.569 (0.06)	0.476 (0.10)	-0.092 (0.09)	57.0 (4.8)	49.7 (10.1)	-7.3 (10.6)
Other cardiovascular problems	12	0.500 (0.08)	0.613 (0.09)	+0.113 (0.11)	55.0 (4.8)	59.2 (9.2)	+4.2 (7.9)
Pneumonia**	91	0.501 (0.03)	0.553 (0.03)	+0.053 (0.03)	53.3 (2.4)	56.4 (3.4)	+3.2 (2.8)
Other respiratory problems	11	0.486 (0.08)	0.681 (0.09)	+0.195 (0.09)	50.5 (5.7)	58.6 (7.5)	+8.2 (8.3)
Acute—Intestinal infection	10	0.487 (0.10)	0.516 (0.09)	+0.029 (0.10)	56.0 (7.3)	48.0 (6.3)	-8.0 (11.2)
Chronic—Intestinal infection	14	0.434 (0.08)	0.400 (0.09)	-0.035 (0.10)	50.0 (2.5)	42.9 (8.4)	-7.1 (8.2)
Upper gastrointestinal disorders	11	0.501 (0.06)	0.485 (0.10)	-0.015 (0.10)	52.5 (3.7)	57.4 (9.9)	+4.9 (10.5)
Liver disease	14	0.436 (0.09)	0.405 (0.10)	-0.032 (0.07)	45.0 (5.9	41.4 (10.3)	-3.6 (8.4)
Diseases of the genitourinary system	18	0.538 (0.06)	0.584 (0.09)	+0.047 (0.08)	53.3 (4.9	59.7 (7.5)	+6.4 (7.1)
Diseases of the musculoskeletal system	6	0.416 (0.09)	0.338 (0.11)	-0.078 (0.12)	47.5 (4.4)	61.7 (6.0)	+14.2 (7.8)
Other problems (<5 cases)	12	0.431 (0.07)	0.542 (0.08)	+0.112 (0.09)	52.5 (3.9)	57.9 (7.2)	+5.4 (7.5)

*Except in Labour

**Except that caused by TB or Cryptococcal

Table 6 shows the costs for participants by their HIV status. The mean total provider cost of admission for participants who were HIV-negative, HIV-positive and not on ART, and HIV-positive on ART, was US$267.07, US$422.90 and US$316.03, respectively. The mean total provider cost of admission for HIV-positive participants was US$74.78 (bootstrap 95%CI: US$25.41-US$124.15) higher than for HIV-negative participants. Amongst HIV-positive participants, the mean total provider cost was US$106.87 (bootstrap 95%CI: US$25.09-US$188.64) lower for those already on ART on admission.

**Table 6:** Costs and health-related quality of life outcomes by HIV status.

				Mean differences (95% CI)**	
		N	Mean (SE)	HIV-positive v HIV-negative	On ART v Not on ART
**Total health provider cost (2014 US$)**	HIV-negative	175	267.07 (20.2)	74.78 (25.41, 124.15)	-106.87 (-188.64, -25.09)
	HIV-positive: not on ART	108	422.90 (40.4)		
	HIV-positive: on ART	339	316.03 (15.7)		
	HIV status unknown	25	135.42 (19.7)		
**Total direct non-medical and indirect cost (2014 US$)**	HIV-negative	175	75.12 (18.7)	21.98 (-21.36, 65.32)	-35.09 (-98.84, 28.66)
	HIV-positive: not on ART	108	123.71 (30.1)		
	HIV-positive: on ART	339	88.62 (13.8)		
	HIV status unknown	25	11.14 (5.3)		
**Total societal cost (2014 US$)**	HIV-negative	175	342.20 (34.0)	96.76 (17.11, 176.40)	-141.95 (-259.17, -24.73)
	HIV-positive: not on ART	108	546.61 (56.0)		
	HIV-positive: on ART	339	404.65 (25.1)		
	HIV status unknown	25	146.55 (21.3)		
*Admission EQ-5D utility score (Zimbabwean tariff)	HIV-negative	174	0.532 (0.02)	-0.066 (-0.114, -0.019)	0.025 (-0.033, 0.082)
	HIV-positive: not on ART	107	0.447 (0.03)		
	HIV-positive: on ART	336	0.472 (0.02)		
	HIV status unknown	23	0.454 (0.07)		
*Admission VAS score	HIV-negative	174	55.1 (1.4)	-3.2 (-6.7, 0.2)	-2.2 (-7.0, 2.5)
	HIV-positive: not on ART	107	53.5 (2.1)		
	HIV-positive: on ART	336	51.3 (1.1)		
	HIV status unknown	23	53.9 (5.8)		
*Admission EQ-5D utility score (UK tariff)	HIV-negative	174	0.335 (0.03)	-0.096 (-0.165, -0.027)	0.058 (-0.022, 0.138)
	HIV-positive: not on ART	107	0.195 (0.03)		
	HIV-positive: on ART	336	0.253 (0.02)		
	HIV status unknown	23	0.280 (0.09)		

ART: Anti-retroviral treatment

*Missing quality of life assessment—HIV negative: 1; HIV positive not on ART: 1; HIV positive on ART: 3; HIV status unknown: 2

**Bootstrapped estimates of mean differences and 95%CI

There were no significant differences in the mean total direct non-medical and indirect cost by HIV or ART status. The mean total societal cost of hospital admission for participants who were HIV-negative, HIV-positive and not on ART, and HIV-positive on ART, was US$342.20, US$546.61 and US$404.65, respectively. The mean total societal cost of admission for HIV-positive participants was US$96.76 (bootstrap 95%CI: US$17.11-US$176.40) higher than for HIV-negative participants. Amongst HIV-positive participants, the mean total societal cost of admission was US$141.95 (bootstrap 95%CI: US$24.73-US$259.17) lower for those already on ART on admission.

The mean admission EQ-5D utility score for participants who were HIV-negative, HIV-positive and not on ART, and HIV-positive on ART, was 0.532, 0.447 and 0.472, respectively (Table 6). The mean admission EQ-5D utility score amongst HIV-negative participants was 0.066 (bootstrap 95%CI: 0.019–0.114) higher than for HIV-positive participants. There were no significant differences in the admission EQ-5D utility scores between HIV-positive participants who were on or not on ART.

In the multivariable analysis (Model 1; Table 7), after adjusting for participant characteristics and discharge medical diagnosis, the mean total provider costs of hospital admission was US$51.04 (95%CI: US$7.23-US$94.86) lower for those who were HIV-positive and on ART on admission compared to those who were HIV-positive and not on ART. There was no significant difference in the mean total provider costs between HIV-negative individuals and HIV-positive individuals not on ART on admission. After adjusting for discharge medical diagnosis (Model 2), we did not find any significant differences in either mean total direct non-medical and indirect costs or mean total societal costs by HIV or ART status.

**Table 7:** Multivariate analysis exploring relationship between HIV and ART status and mean total costs*.

	Total health provider cost (2014 US Dollars)		Total direct non-medical and indirect cost (2014 US Dollars)		Total societal cost (2014 US Dollars)	
	Model 1 (n = 605) Coef (95% CI)	Model 2 (n = 605) Coef (95% CI)	Model 1 (n = 605) Coef (95% CI)	Model 2 (n = 605) Coef (95% CI)	Model 1 (n = 605) Coef (95% CI)	Model 2 (n = 605) Coef (95% CI)
**HIV-positive: not on ART**	Ref	Ref	Ref	Ref	Ref	Ref
**HIV-positive: on ART**	-87.06** (-163.07, -11.06)	-51.04** (-94.86, -7.23)	15.00 (-25.27, 55.26)	18.92 (-23.65, 61.49)	-70.98 (-159.24, 17.27)	-45.99 (-99.88, 7.91)
**HIV-negative**	-140.43** (-219.41, -61.44)	-45.60 (-95.65, 4.45)	-3.14 (-56.17, 49.88)	24.19 (-36.02, 84.40)	-128.65** (-224.49, -32.82)	-34.76 (-99.64, 30.11)
**HIV status unknown**	-279.86** (-361.81, -197.91)	-146.93** (-202.61, -91.26)	-60.23** (-111.56, -8.90)	-9.10 (-68.68, 50.49)	-301.34** (-401.06, -201.63)	-160.95** (-235.91, -85.99)

ART: Anti-retroviral treatment

Model 1: age and sex

Model 2: additionally adjusted for primary medical diagnosis, marital status, educational attainment, income and wealth quintile

*Findings from Generalized linear model with Poisson distribution and identity link function

** p<0.05

The findings of the multivariable analysis exploring the relationship between HIV status and the EQ-5D utility scores on admission are shown in Table 8. In the multivariable analysis, the model diagnostics showed that the OLS estimator performed as well or better than the other estimators (S6 and S7 Tables). In the multivariable analysis, after adjusting for individual characteristics and the discharge medical diagnosis, the mean admission EQ-5D utility score amongst those who were HIV-negative was 0.131 (95%CI: 0.064–0.198) higher than amongst those who were HIV-positive and not on ART on admission. There were no significant differences in the adjusted EQ-5D utility scores between those who were HIV-positive and either taking or not taking ART on admission.

**Table 8:** Multivariate analysis exploring relationship between HIV and ART status and health-related quality of life outcomes on admission*.

	Admission EQ-5D utility score (Zimbabwean tariff)		Admission VAS score		Admission EQ-5D utility score (UK tariff)	
	Model 1 (n = 605) Coef (95% CI)	Model 2 (n = 605) Coef (95% CI)	Model 1 (n = 605) Coef (95% CI)	Model 2 (n = 605) Coef (95% CI)	Model 1 (n = 605) Coef (95% CI)	Model 2 (n = 605) Coef (95% CI)
**HIV-positive: not on ART**	Ref	Ref	Ref	Ref	Ref	Ref
**HIV-positive: on ART**	0.038 (-0.019, 0.095)	0.048 (-0.011, 0.106)	-0.99 (-5.23, 3.25)	-0.55 (-4.80, 3.69)	0.070 (-0.013, 0.152)	0.085 (-0.001, 0.170)
**HIV-negative**	0.112** (0.051, 0.173)	0.131** (0.064, 0.198)	2.92 (-1.63, 7.47)	3.20 (-1.60, 7.99)	0.185** (0.095, 0.275)	0.207** (0.108, 0.306)
**HIV status unknown**	0.076 (-0.066, 0.218)	0.092 (-0.062, 0.245)	7.85 (-0.85, 16.54)	9.47 (-0.07, 19.01)	0.147 (-0.062, 0.356)	0.160(-0.065, 0.385)

Model 1: age and sex

Model 2: additionally adjusted for primary medical diagnosis, marital status, educational attainment, income and wealth quintile

*Findings from ordinary least squares estimator

** p<0.05

## Discussion

The main findings of this study are the high costs incurred in managing adults admitted to hospital in a resource-poor setting with high HIV prevalence. Health provider costs were especially high for managing HIV-associated illnesses. However, costs were substantially lower, with significantly shorter duration of admission and less risk of death, if individuals were already receiving ART on hospital admission. Health-related quality of life was especially poor amongst those admitted for HIV-associated illnesses, and overall, was significantly lower in HIV-positive than HIV-negative participants. Our data also highlights the substantial burden imposed on the finances of patients and their families as a result of hospitalisation. Even though patients do not pay for medical services, the mean cost from the patient perspective was US$87.84, amounting to catastrophic costs for most patients.

In this study, the average health provider cost of managing individuals in hospital was US$313.65, with substantially higher costs for HIV-positive individuals and for AIDS-defining diseases. Total provider costs of one year of ART have been estimated to be US$136 (in 2011 prices) in Malawi [36]. HIV-positive individuals will continue to be at increased risk of hospitalisation after initiation of ART, especially if treatment is started late, but at a population level, timely ART initiation will reduce the absolute numbers requiring admission [37]. Moreover, the substantial cost differences found between those taking and not taking ART in this study raise the prospect of considerably higher savings from ART than would be anticipated on the basis of admission rates alone. This difference remained even after accounting for differences in cause of admission. Thus, the costs incurred in providing early initiation of ART to greater numbers of people living with HIV may be offset by larger cost savings than have been appreciated. Importantly, we found that the majority of patients were aware of their HIV status, and many of those who were HIV-positive had already started ART. In the study we were unable to ascertain the stage of participants’ HIV infection, or when ART was initiated.

In Malawi, hospital care is provided free but users inevitably incur some costs in accessing care, including for transportation to and from hospital, and losses in income. The mean direct non-medical and indirect cost was estimated at US$86.93. The majority of Malawians live on less than $2 a day [38], highlighting the catastrophic impact of a hospitalisation on the finances of Malawians. Whilst preventing illness will have a major impact on reducing this burden, offering social security benefits to those affected needs to be explored further [39].

Tuberculosis continues to be one of the most common reasons for medical inpatient care in sub-Saharan Africa [1]. As in previous studies and surveillance data, the majority of TB patients in our study were HIV positive. Individuals with HIV and TB coinfection reported very poor HRQoL, with hospitalisation resulting in substantial costs for them and for the health system. Hospitalisation may be unavoidable for some TB patients, considering the severity of their illness, but moving the later stages of care to community-based TB services, which are already established in much of the region, could reduce these costs [40]. Rapid scale up TB preventive therapy and systematic TB screening on all health encounters are urgently required [41].

The World Health Organization’s (WHO) prequalification of medicines programme ensures high quality drugs enter African healthcare markets at reasonable prices [42]. In our study, we found drugs accounted for a lower proportion of total health provider costs than investigations and procedures. The WHO prequalification programme does extend to diagnostics and medical devices; however, the focus has predominantly been around rapid diagnostic and point of care tests. Extending these services to all medical consumables and equipment may reduce costs, and facilitate decentralisation of diagnostic services to district level hospitals.

We used the EuroQol EQ-5D measure to provide two assessments of HRQoL, including one (EQ-5D utility score) that can be used to inform cost-utility analyses. The mean EQ-5D utility score reported was 0.498, with HIV-positive inpatients reporting much lower HRQoL than HIV-negative inpatients. The mean EQ-5D utility score amongst HIV-positive inpatients who had not started ART (0.447) was considerably lower than reported by HIV-positive outpatients in the catchment population of this hospital (0.8) [21]. This further reinforces the value of early diagnosis and ART initiation to prevent serious and debilitating illness, and maintenance of HRQoL. Of concern were the minimal changes in HRQoL outcomes during admission, although this has to be interpreted in the context of high inpatient mortality. Health utility data are notably lacking in this region, constraining the use of cost-utility analyses in economic evaluations [43, 44]. This study provides an extensive catalogue of health utility scores, including those derived using an alternative tariff set (UK York A1), to inform cost-utility analyses for a range of interventions, not just limited to HIV.

Study limitations include the relatively small number of participants recruited for a few of the medical conditions; discharge medical diagnoses based on the assessment of one medical doctor; and the fact the study was undertaken in a large central teaching hospital that limits generalisability to smaller district hospital settings. In addition, we were unable to examine economic outcomes in the sickest group of patients, those who died before recruitment was possible, or whose medical notes were not found. However, this is the first study we are aware of that estimates economic costs and HRQoL outcomes amongst a cohort of adults admitted to hospital for medical reasons in an African context with high HIV prevalence. We collected individual-level data on healthcare resources used, direct non-medical and indirect costs incurred, and examined HRQoL outcomes. We undertook a primary costing study to estimate the costs of all healthcare resources used, and provide estimates of the total health provider costs.

Our findings highlight the catastrophic costs and poor HRQoL outcomes associated with hospitalisation in a sub-Saharan cohort with high HIV prevalence. Importantly, as countries in sub-Saharan Africa move towards immediate initiation of ART treatment for people living with HIV, policy makers will need to be aware of the potential for substantial cost savings from averting serious HIV-associated illnesses and through earlier case detection of tuberculosis.

## Supporting information

S1 Text Direct health provider costing methods. (DOCX)

S2 Text Direct non-medical and indirect costing methods. (DOCX)

S3 Text Health-related quality of life assessment methods. (DOCX)

S1 Table Mean health provider unit cost—Ward stay and drug dispensing costs. (DOCX)

S2 Table Mean health provider unit cost—Radiological and imaging investigations. (DOCX)

S3 Table Mean health provider unit cost—Laboratory investigations. (DOCX)

S4 Table Mean health provider unit cost—Ward-based investigations and procedures. (DOCX)

S5 Table EQ-5D utility scores (UK tariff) by discharge medical diagnosis. (DOCX)

S6 Table Estimated predicted values compared to actual utility scores. (DOCX)

S7 Table MSE, MAE and R-squared statistics for regression models by utility score range. (DOCX)
