# Supplementary material for: Economic costs and health-related quality of life outcomes of hospitalised patients with high HIV prevalence: A prospective hospital cohort study in Malawi
Source: PLoS One. 2018 Mar 15;13(3):e0192991. doi: 10.1371/journal.pone.0192991 (PMC5854246; doi:10.1371/journal.pone.0192991)
Supplement: S1 Text — (DOCX) [file pone.0192991.s001.docx]

**S1 Text: Direct health provider costing methods**

UNAIDS costing guidelines were used to undertake the primary costing studies to estimate the costs for all medical resource outputs used [1]. Broadly, a list of medical resource outputs (e.g. days of admission; full blood count) was identified from the medical data extracted by the doctors. Secondly, interviews were conducted with medical and administrative personnel to identify and quantify the individual resources required to produce the medical resource outputs. Thirdly, the financial data from hospital administration systems, in combination with National and International resource data, were used to value individual resource inputs. Fourthly, the data was used to estimate the cost of each medical resource output. Finally, the estimated resource output costs were used to estimate the total health provider cost for each study participant. A combination of top-down and bottom-up methods were used to estimate the relevant costs [2].

We undertook interviews with central support services to estimate the total central support service costs for running the hospital. This cost was then allocated to each department (Medical wards, Laboratory, Radiology and Pharmacy). We used the number of staff working in each of these departments divided by the total number of staff at the hospital to estimate the proportion of the total central support costs consumed by each relevant department. Some central support costs (e.g. catering, costs of locum staff) were only allocated to the medical wards, as the other departments do not benefit from them. For this we used the number of clinical staff working on the medical wards and divided by the total number of clinical staff working in the hospital to determine what proportion of the specific form of central support cost should be allocated to each medical ward.

For the medical wards, we estimated the cost per day of hospital care. Interviews were undertaken with the nurse in charge on the wards to determine the staff who worked on the ward and the time they spent on the ward. Information was obtained on the consumables used, and the quantity of each consumable used annually. A list of all the equipment on the wards was also documented. A unit cost was obtained for all consumables and equipment’s. The total average annual cost of running the ward was estimated. This compromised the cost of staff, consumables and equipment. The cost of central support services was added to these total costs. During the study period, we recorded the daily number of patients on each ward, and this was used to estimate the total number of patient days of admission per year. The total average annual cost for each ward was divided by annual patient days to estimate the average cost per patient day of admission.

We repeated the same procedure for the pharmacy department. We estimated the total average annual cost of running the pharmacy. This compromised the costs of staff, consumables and equipment, in addition to an allocated proportion of the central support services. The pharmacy keeps a record of the total drugs supplied per annum, broken down by drug formulation and doses given. The total average annual cost of running the pharmacy was divided by the total number of drug doses supplied to estimate the average cost per dose of drug dispensed by the pharmacy. This cost was added to the cost of the drug to estimate the total cost of providing the drug to the patient. For the cost of the drugs, we used the international market price [3]. The cost of shipping and insurance was excluded from the international market price for the drugs.

Investigations and procedures are performed through the Laboratory and Radiology department at QECH or on the medical wards. For investigations and procedures, we estimated the direct cost of performing the investigation and the indirect cost of the investigation or procedure. The direct cost of the investigation or procedure comprised the attributable cost of personnel, consumables and equipment. We undertook interviews with the medical, laboratory and radiology staff to quantify each of these items. For equipment and personnel, we recorded the approximate time spent in performing the investigation. A unit cost of each item was obtained and the total direct cost of the investigation or procedure was estimated. The indirect cost of the investigation comprised the cost per test of the department and the cost of the central support services. For this we estimated total cost, including the cost of central support services, and divided this by the total outputs of the department. For investigations and procedures performed in the Laboratory and Radiology department, we used the total number of tests performed as the denominator. For the Laboratory and Radiology department, we undertook interviews to estimate the numbers and grades of staff working in the department, and the consumables and equipment used. For all these items we quantified the proportion of the time spent on performing investigations or procedures, and the proportion of time spent on general duties or activities. For the indirect costs, we summed total costs based on resources used for activities other than performing a specific investigation or procedure. We used the estimated direct cost of performing investigations and procedures performed on the ward. Some investigations are performed outside QECH. For these we used the cost charged to QECH as the total cost of the investigation.

For all the costings, we obtained staff salaries from the QECH Human Resources departments and included employer contributions and fringe benefits. The costs of consumables and equipment were obtained from the Malawi Ministry of Health price catalogue. For costs that were not available in the catalogue, we used the international market prices. We first contacted local suppliers to obtain costs, and if these were not available, we contacted international suppliers and manufacturers. Equipment costs were annuitized over their useful life with an annual discount rate of 3%. For large laboratory and imaging equipment, we assumed the useful lifetime to be 5 years. This assumption was based on discussions with laboratory and radiology staff on time before equipment should be replaced. For all other equipment, including general office equipment, we assumed the useful lifetime to be 3 years.

The *total direct health provider cost* comprised the cost of the stay on the hospital ward, the cost of all investigations and procedures and the cost of all drugs given. The cost of stay on the hospital ward was estimated by multiplying the average cost per day of admission by the total number of days stayed on each ward. The total cost of investigations and procedures per participant was estimated by multiplying the relevant unit costs by the number of times the investigations and procedures were performed. The total cost of drugs per participant was estimated by multiplying the cost of each drug given by the number of doses administered. The cost of each drug included the cost of dispensing the drug through the pharmacy department.

**References**

1. UNAIDS. Manual for costing HIV facilities and services. Available at: <http://www.unaids.org/sites/default/files/en/media/unaids/contentassets/documents/document/2011/20110523_manual_costing_HIV_facilities_en.pdf> (Accessed May 2014). **2011**.

2. Drummond MF, Sculpher MJ, Torrance GW. Methods for the economic evaluation of health care programmes: Oxford University Press, USA, **2005**.

3. Health MSf. International Drug Price Indicator Guide. Available at: https://<http://www.msh.org/blog/2014/07/30/2013-international-drug-price-indicator-guide-now-available>. **2013**.

4. Pritchard C, Sculpher M. Productivity costs: principles and practice in economic evaluation. Available at: https://<http://www.ohe.org/publications/productivity-costs-principles-and-practice-economic-evaluation> (Accessed May 2015): Office of Health Economics London, **2000**.

5. Dolan P, Gudex C, Kind P, Williams A. The time trade-off method: results from a general population study. Health economics **1996**; 5(2): 141-54.

6. Jelsma J, Hansen K, De Weerdt W, De Cock P, Kind P. How do Zimbabweans value health states? Popul Health Metr **2003**; 1(1): 11.
