# Supplementary material for: Economic costs and health-related quality of life outcomes of hospitalised patients with high HIV prevalence: A prospective hospital cohort study in Malawi
Source: PLoS One. 2018 Mar 15;13(3):e0192991. doi: 10.1371/journal.pone.0192991 (PMC5854246; doi:10.1371/journal.pone.0192991)
Supplement: S2 Text — (DOCX) [file pone.0192991.s002.docx]

**S2 Text: Direct non-medical and indirect costing methods**

Two interviewer-administered questionnaires were developed to record direct non-medical and indirect costs incurred by participants and their main family member or carer who remained with them at hospital during the admission. The direct non-medical costs included the cost of transportation, food, drinks, toiletries, clothing and other items bought during the hospital admission. For indirect costs, we recorded whether participants or their carers had taken time off work, and if so, the amount of time, and multiplied this by their self-reported income [1]. The first questionnaire was administered on the first working day after hospital admission, and asked about all direct non-medical and indirect costs incurred on the day of their hospital admission. Follow-up questionnaires were administered to participants every three to seven days thereafter, and captured direct non-medical and indirect costs for the preceding day they were in hospital.

The *total direct non-medical and indirect cost* per participant was estimated for the duration of the hospital admission. This included costs incurred by the participant and their main family member/carer who stayed with them during their hospital admission. For participants who died during their hospital admission, these costs were estimated for the period from admission till death. The total direct non-medical and indirect cost was estimated by adding the costs on the day of admission, to the average daily cost for each subsequent period between interviews multiplied by the duration of each subsequent period.

The questionnaires were forward translated into Chichewa, the local language of the study population, and back translated by two independent bilingual Malawians. The questionnaires were then pilot tested, and discussions were held with senior Malawian staff working at the Malawi-Liverpool Wellcome Trust Clinical Research Programme before the final version was agreed upon.

**References**

1. Pritchard C, Sculpher M. Productivity costs: principles and practice in economic evaluation. Available at: <https://www.ohe.org/publications/productivity-costs-principles-and-practice-economic-evaluation> (Accessed May 2015): Office of Health Economics London; 2000.
