# Supplementary material for: Economic costs and health-related quality of life outcomes of hospitalised patients with high HIV prevalence: A prospective hospital cohort study in Malawi
Source: PLoS One. 2018 Mar 15;13(3):e0192991. doi: 10.1371/journal.pone.0192991 (PMC5854246; doi:10.1371/journal.pone.0192991)
Supplement: S3 Text — (DOCX) [file pone.0192991.s003.docx]

**S3 Text: Health-related quality of life assessment methods**

All study participants were asked about their health-related quality of life (HRQoL) on the first day after their admission or on the first day on which they were able to respond to the questions. HRQoL was assessed every three to seven days thereafter, however, we examined the earliest recorded assessment in order to reflect the pre-treatment health state.

Participants completed both the descriptive EQ-5D-3L system and the visual analogue scale (VAS). The responses to the descriptive component were converted to an EQ-5D utility score by using a tariff set. The tariff sets have been derived from national surveys of the general population, with a subset of the 243 health states being valued, most commonly using the time trade-off method [1]. The remainder of the EQ-5D health states were subsequently valued through the estimation of a multivariate model. As there is no Malawian EQ-5D tariff, the Zimbabwean EQ-5D tariff set [2] was used. The visual analogue scale is similar to a thermometer, and ranges from 100 (best imaginable health state) to 0 (worst imaginable health state). Participants recorded how good or bad their health was on the day of completion by drawing a line on the scale.

**References**

1. Dolan P, Gudex C, Kind P, Williams A. The time trade-off method: results from a general population study. Health economics. 1996;5(2):141-54. Epub 1996/03/01. doi: 10.1002/(SICI)1099-1050(199603)5:2<141::AID-HEC189>3.0.CO;2-N [pii]

10.1002/(SICI)1099-1050(199603)5:2<141::AID-HEC189>3.0.CO;2-N. PubMed PMID: 8733106.

2. Jelsma J, Hansen K, De Weerdt W, De Cock P, Kind P. How do Zimbabweans value health states? Popul Health Metr. 2003;1(1):11. Epub 2003/12/18. doi: 10.1186/1478-7954-1-11

1478-7954-1-11 [pii]. PubMed PMID: 14678566; PubMed Central PMCID: PMC317383.
