# Supplementary material for: Economic costs and health-related quality of life outcomes of hospitalised patients with high HIV prevalence: A prospective hospital cohort study in Malawi
Source: PLoS One. 2018 Mar 15;13(3):e0192991. doi: 10.1371/journal.pone.0192991 (PMC5854246; doi:10.1371/journal.pone.0192991)
Supplement: S1 Table — (DOCX) [file pone.0192991.s004.docx]

**S1 Table: Mean health provider unit cost - Ward stay and drug dispensing costs**

| Cost category | Mean direct health provider cost | |
| --- | --- | --- |
|  | 2014 US Dollars | 2014 INT Dollars |
| Cost per day of admission  TB Ward  Male medical  Female medical | 14.48  15.35  16.17 | 40.17  42.58  44.80 |
| Pharmacy dispensing cost  Mean cost per table, vial, ampoule dispensed | 0.0058 | 0.0162 |
