# Supplementary material for: Economic costs and health-related quality of life outcomes of hospitalised patients with high HIV prevalence: A prospective hospital cohort study in Malawi
Source: PLoS One. 2018 Mar 15;13(3):e0192991. doi: 10.1371/journal.pone.0192991 (PMC5854246; doi:10.1371/journal.pone.0192991)
Supplement: S2 Table — (DOCX) [file pone.0192991.s005.docx]

**S2 Table: Mean health provider unit cost - Radiological and imaging investigations**

| Investigation | Mean Total Cost | |
| --- | --- | --- |
|  | 2014 US Dollars | 2014 INT Dollars |
| Chest X-Ray | 10.73 | 27.56 |
| Abdominal X-Ray | 10.73 | 27.56 |
| Cervical Spine X-Ray | 7.85 | 21.78 |
| Thoracic Spine X-Ray | 7.85 | 21.78 |
| Lumbar Spine X-Ray | 7.85 | 21.78 |
| Other plain X-Ray | 10.73 | 27.56 |
| Abdominal/Renal Ultrasound | 16.81 | 46.67 |
| Pelvic Ultrasound | 16.81 | 46.67 |
| Neck Ultrasound | 16.81 | 46.67 |
| Doppler Ultrasound | 19.25 | 53.45 |
| Chest Ultrasound | 16.81 | 46.67 |
| *MRI Head | 50.89 | 68.55 |
| *MRI Spine | 50.89 | 68.55 |
| *CT Head | 22.08 | 61.33 |
| *CT Thorax | 22.08 | 61.33 |
| *CT Abdomen | 22.08 | 61.33 |

*Service out-sourced to external provider

MRI: Magnetic resonance imaging

CT: Computed Tomography
