# Supplementary material for: Economic costs and health-related quality of life outcomes of hospitalised patients with high HIV prevalence: A prospective hospital cohort study in Malawi
Source: PLoS One. 2018 Mar 15;13(3):e0192991. doi: 10.1371/journal.pone.0192991 (PMC5854246; doi:10.1371/journal.pone.0192991)
Supplement: S3 Table — (DOCX) [file pone.0192991.s006.docx]

**S3 Table: Mean health provider unit cost - Laboratory investigations**

| Investigation +/- Procedure | Mean Total Cost | |
| --- | --- | --- |
|  | 2014 US Dollars | 2014 INT Dollars |
| Malaria Film | 2.56 | 7.11 |
| Peripheral blood film | 4.93 | 13.43 |
| Group and X match | 13.22 | 36.86 |
| Full Blood Count (FBC) | 5.35 | 14.28 |
| Erythrocyte sedimentation rate (ESR) | 1.47 | 4.08 |
| *Prothrombin Time | 12.24 | 34.00 |
| Urea + Electrolytes (U+Es) | 11.33 | 31.56 |
| Creatinine | 3.94 | 11.04 |
| Liver Function Tests (LFTs) | 8.76 | 24.44 |
| Lipid Profile | 10.44 | 29.09 |
| Cardiac enzymes | 8.19 | 22.83 |
| Random / Fasting glucose | 3.69 | 10.36 |
| CD4 count | 15.48 | 42.99 |
| HIV Viral Load | 27.63 | 31.04 |
| Hepatitis B surface antigen (Hep B sAg) | 3.32 | 9.22 |
| Hepatitis C antibody (Hep C Ab) | 5.88 | 9.51 |
| VDRL | 3.35 | 9.31 |
| Malaria rapid diagnostic test | 2.16 | 6.00 |
| Blood Culture | 33.77 | 82.18 |
| Urine microscopy | 2.40 | 6.70 |
| Stool microscopy | 2.10 | 5.85 |
| CSF/LP | 41.63 | 114.27 |
| Sputum smear (Microscopy and AFB) | 5.86 | 15.01 |
| Sputum for GeneXpert (rapid TB test) | 25.51 | 60.69 |
| *Sputum culture for Tuberculosis | 17.42 | 48.39 |
| Lymph node aspirate for Micro (AFB, cell count) | 7.76 | 20.21 |
| *Lymph node aspirate for Cytology | 29.87 | 82.98 |
| Lymph node biopsy for Micro (AFB) | 6.77 | 17.57 |
| Lymph node biopsy for Histology | 49.53 | 136.63 |
| Cytology | 27.66 | 76.84 |
| Pregnancy Test | 3.93 | 10.94 |
| **Diagnostic Pleural Tap | 17.60 | 47.73 |
| **Diagnostic Ascitic Tap | 17.60 | 47.73 |
| **Therapeutic and Diagnostic Ascitic Tap | 23.60 | 64.40 |
| **Diagnostic Knee Aspirate | 16.06 | 43.45 |
| **Therapeutic and Diagnostic Pleural Tap | 23.60 | 64.40 |

*Service out-sourced to external provider

**Includes cost of procedure

Urea + Electrolytes (U+Es): Urea, Sodium and Potassium

Liver Function Tests (LFTs): Total protein, Albumin, Bilirubin, Aspartate aminotransferase (AST), Alanine aminotransferase (ALT) Gamma-glutamyl transpeptidase (GGT) and Lactate dehydrogenase (LDH)

Lipid Profile: Total Cholesterol (TC), Triglyceride (TG), Low-density lipoprotein (LDL), high-density lipoprotein (HDL)

Cardiac enzymes: Creatine Kinase (CK & CKMD), Lactate dehydrogenase (LD)

VDRL: Venereal Disease Research Laboratory test for Syphilis

CSF/LP: Lumbar puncture to obtain cerebrospinal fluid

AFB: Acid-fast bacilli test for Tuberculosis
