# Supplementary material for: Economic costs and health-related quality of life outcomes of hospitalised patients with high HIV prevalence: A prospective hospital cohort study in Malawi
Source: PLoS One. 2018 Mar 15;13(3):e0192991. doi: 10.1371/journal.pone.0192991 (PMC5854246; doi:10.1371/journal.pone.0192991)
Supplement: S4 Table — (DOCX) [file pone.0192991.s007.docx]

**S4 Table: Mean health provider unit cost - Ward-based investigations and procedures**

| Investigation or Procedure | Mean Total Cost | |
| --- | --- | --- |
|  | 2014 US Dollars | 2014 INT Dollars |
| Urine Dipstick | 0.82 | 2.27 |
| HIV Test | 3.87 | 10.74 |
| Electrocardiography (ECG) | 2.61 | 7.24 |
| Echocardiogram | 15.05 | 41.82 |
| Therapeutic Pleural Tap | 12.88 | 35.78 |
| Therapeutic Ascitic Tap | 4.55 | 12.63 |
| Lymph node aspirate | 2.21 | 6.14 |
| Insertion of Naso-Gastric tube | 3.87 | 10.74 |
| Insertion urinary catheter | 11.65 | 32.37 |
| Incision and drainage | 3.40 | 9.44 |
| Chest Drain | 32.71 | 88.63 |
| Therapeutic Lumbar Puncture | 8.55 | 22.13 |
| *Gastroscopy/Endoscopy/Laryngoscopy | 470.70 | 1307.51 |
| *Colonoscopy | 470.70 | 1307.51 |
| *Bronchoscopy | 470.70 | 1307.51 |
| *Endoscopy +/- Banding | 470.70 | 1307.51 |
| Laparoscopic Surgery | Not costed | |
| Laparotomy | Not costed | |

*Not costed, cost obtained from private health provider
