# Supplementary material for: Economic costs and health-related quality of life outcomes of hospitalised patients with high HIV prevalence: A prospective hospital cohort study in Malawi
Source: PLoS One. 2018 Mar 15;13(3):e0192991. doi: 10.1371/journal.pone.0192991 (PMC5854246; doi:10.1371/journal.pone.0192991)
Supplement: S5 Table — (DOCX) [file pone.0192991.s008.docx]

**S5 Table: EQ-5D utility scores (UK tariff) by discharge medical diagnosis**

| Discharge medical diagnosis | N | EQ-5D utility scores (UK tariff) | | |
| --- | --- | --- | --- | --- |
|  |  | **On admission** | **Last recorded** | **Change** |
|  |  | Mean (SE) | Mean (SE) | Mean (SE) |
| All | **640** | **0.267 (0.02)** | **0.382 (0.02)** | **+0.116 (0.02)** |
| Pulmonary tuberculosis | 54 | 0.207 (0.05) | 0.391 (0.05) | +0.184 (0.05) |
| Tuberculosis of meninges and central nervous system | 16 | 0.062 (0.10) | 0.192 (0.07) | +0.130 (0.14) |
| Tuberculosis of intestines, peritoneum | 9 | 0.384 (0.14) | 0.332 (0.10) | -0.053 (0.09) |
| Tuberculosis of bones and joint | 4 | 0.073 (0.15) | 0.125 (0.07) | +0.052 (0.11) |
| Tuberculosis of other organs | 15 | 0.324 (0.10) | 0.309 (0.10) | -0.015 (0.09) |
| Miliary tuberculosis | 17 | 0.156 (0.10) | 0.074 (0.06) | -0.082 (0.09) |
| Tuberculosis - retreatment | 6 | 0.412 (0.22) | 0.551 (0.18) | +0.139 (0.10) |
| Septicaemia* | 58 | 0.303 (0.05) | 0.474 (0.05) | +0.171 (0.05) |
| Candidiasis | 6 | 0.073 (0.07) | 0.207 (0.11) | +0.134 (0.15) |
| Cryptococcal meningitis | 36 | 0.236 (0.07) | 0.348 (0.07) | +0.112 (0.08) |
| Viral infection | 8 | 0.449 (0.10) | 0.361 (0.15) | -0.088 (0.09) |
| Pneumocystis Jivorecii pneumonia | 8 | 0.382 (0.13) | 0.392 (0.17) | +0.010 (0.21) |
| Malaria | 13 | 0.338 (0.09) | 0.326 (0.11) | -0.011 (0.03) |
| Kaposi’s sarcoma | 20 | 0.145 (0.07) | 0.228 (0.07) | +0.083 (0.05) |
| Neoplasms - excluding Kaposi's | 7 | 0.424 (0.14) | 0.149 (0.20) | -0.276 (0.18) |
| Diabetes mellitus without complications | 5 | 0.610 (0.09) | 0.791 (0.06) | +0.181 (0.12) |
| Diabetes mellitus with complications | 9 | 0.148 (0.14) | 0.186 (0.14) | +0.038 (0.09) |
| Anaemia | 35 | 0.355 (0.07) | 0.492 (0.07) | +0.137 (0.06) |
| Mental health disorders | 9 | 0.426 (0.12) | 0.564 (0.12) | +0.137 (0.15) |
| Meningitis** | 36 | 0.294 (0.06) | 0.518 (0.07) | +0.225 (0.07) |
| Epilepsy; Convulsions | 10 | 0.381 (0.18) | 0.437 (0.20) | +0.056 (0.09) |
| Other neurological problems | 15 | 0.308 (0.10) | 0.368 (0.10) | +0.060 (0.09) |
| Cerebrovascular disease | 23 | 0.087 (0.08) | 0.224 (0.10) | +0.138 (0.06) |
| Hypertension | 7 | 0.132 (0.19) | 0.245 (0.15) | +0.114 (0.13) |
| Congestive heart failure; non-hypertensive | 15 | 0.371 (0.10) | 0.416 (0.10) | +0.045 (0.10) |
| Other cardiovascular problems | 12 | 0.308 (0.12) | 0.536 (0.10) | +0.228 (0.13) |
| Pneumonia** | 91 | 0.295 (0.04) | 0.434 (0.04) | +0.139 (0.04) |
| Other respiratory problems | 11 | 0.236 (0.12) | 0.609 (0.11) | +0.373 (0.13) |
| Acute - Intestinal infection | 10 | 0.292 (0.15) | 0.332 (0.11) | +0.039 (0.14) |
| Chronic - Intestinal infection | 14 | 0.170 (0.12) | 0.274 (0.11) | +0.104 (0.11) |
| Upper gastrointestinal disorders | 11 | 0.286 (0.10) | 0.331 (0.12) | +0.045 (0.13) |
| Liver disease | 14 | 0.229 (0.11) | 0.306 (0.10) | +0.077 (0.09) |
| Diseases of the genitourinary system | 18 | 0.307 (0.08) | 0.491 (0.10) | +0.184 (0.11) |
| Diseases of the musculoskeletal system | 6 | 0.131 (0.10) | 0.031 (0.13) | -0.100 (0.14) |
| Other problems (<5 cases) | 12 | 0.170 (0.10) | 0.370 (0.11) | +0.200 (0.10) |

*Except in Labour

**Except that caused by TB or Cryptococcal
