# Supplementary material for: Economic costs and health-related quality of life outcomes of hospitalised patients with high HIV prevalence: A prospective hospital cohort study in Malawi
Source: PLoS One. 2018 Mar 15;13(3):e0192991. doi: 10.1371/journal.pone.0192991 (PMC5854246; doi:10.1371/journal.pone.0192991)
Supplement: S6 Table — (DOCX) [file pone.0192991.s009.docx]

S6 Table: Estimated predicted values compared to actual utility scores

|  | **Model** | **Obs** | **Mean** | **Min** | **Max** | **MSE** | **MAE** | **r^2^** |
| --- | --- | --- | --- | --- | --- | --- | --- | --- |
|  | Observed | 605 | 0.503 | -0.145 | 1.000 |  |  |  |
| Model | OLS | 605 | 0.503 | 0.242 | 0.772 | 0.000 | 0.199 | 0.119 |
|  | TOBIT | 605 | 0.502 | 0.241 | 0.771 | 0.001 | 0.200 | 0.118 |
|  | CLAD | 605 | 0.519 | 0.095 | 0.985 | 0.017 | 0.206 | 0.046 |
|  | Flogit | 605 | 0.503 | 0.242 | 0.772 | 0.000 | 0.199 | 0.119 |

OLS: Ordinary Least Squares MSE: Mean Squared Error

Flogit: Fractional logit MAE: Mean Absolute Error

CLAD: Censored least absolute deviations r^2^: Coefficient of determination
