# Supplementary material for: Economic costs and health-related quality of life outcomes of hospitalised patients with high HIV prevalence: A prospective hospital cohort study in Malawi
Source: PLoS One. 2018 Mar 15;13(3):e0192991. doi: 10.1371/journal.pone.0192991 (PMC5854246; doi:10.1371/journal.pone.0192991)
Supplement: S7 Table — (DOCX) [file pone.0192991.s010.docx]

S7 Table: MSE, MAE and R-squared statistics for regression models by utility score range

|  |  |  | |  | |  | |  | |  | | **Observed EQ-5D utility score** | | | | | | | | | | | | | | | |  |
| --- | --- | --- | --- | --- | --- | --- | --- | --- | --- | --- | --- | --- | --- | --- | --- | --- | --- | --- | --- | --- | --- | --- | --- | --- | --- | --- | --- | --- |
|  | <0 | | | | | | 0 to <0·2 | | | | | | 0·2 to <0·4 | | | 0·4 to <0·6 | | | 0·6 to <0·8 | | | 0·8 to <1 | | | 1 | | | |
| **Obs** | 29 | | | | | | 45 | | | | | | 139 | | | 155 | | | 180 | | | 56 | | | 14 | | | |
|  | MSE | | MAE | | r^2^ | | MSE | | MAE | | r^2^ | | MSE | MAE | r^2^ | MSE | MAE | r^2^ | MSE | MAE | r^2^ | MSE | MAE | r^2^ | MSE | MAE | r^2^ | |
| **OLS** | 0.495 | | 0.495 | | 0.018 | | 0.355 | | 0.355 | | 0.012 | | 0.180 | 0.180 | 0.043 | 0.003 | 0.079 | 0.008 | 0.178 | 0.179 | 0.014 | 0.305 | 0.305 | 0.008 | 0.433 | 0.433 | n/a | |
| **TOBIT** | 0.495 | | 0.495 | | 0.016 | | 0.354 | | 0.354 | | 0.010 | | 0.179 | 0.180 | 0.040 | 0.004 | 0.078 | 0.009 | 0.179 | 0.180 | 0.014 | 0.308 | 0.308 | 0.010 | 0.426 | 0.426 | n/a | |
| **CLAD** | 0.502 | | 0.502 | | 0.021 | | 0.407 | | 0.407 | | 0.005 | | 0.176 | 0.193 | 0.015 | 0.008 | 0.104 | 0.002 | 0.157 | 0.168 | 0.024 | 0.261 | 0.268 | 0.001 | 0.421 | 0.421 | n/a | |
| **Flogit** | 0.495 | | 0.495 | | 0.018 | | 0.355 | | 0.355 | | 0.012 | | 0.180 | 0.180 | 0.043 | 0.003 | 0.079 | 0.008 | 0.178 | 0.179 | 0.014 | 0.305 | 0.305 | 0.008 | 0.433 | 0.433 | n/a | |

OLS: Ordinary Least Squares Flogit: Fractional logit CLAD: Censored least absolute deviations

MSE: Mean Squared Error MAE: Mean Absolute Error r^2^: Coefficient of determination
